# Stunning of common carp: Results from a field and a laboratory study

**DOI:** 10.1186/s12917-018-1530-0

**Published:** 2018-06-27

**Authors:** Karina Retter, Karl-Heinz Esser, Matthias Lüpke, John Hellmann, Dieter Steinhagen, Verena Jung-Schroers

**Affiliations:** 1 Fish Disease Research Unit, Institute for Parasitology, University of Veterinary Medicine, Bünteweg 17, D-30559 Hannover, Germany; 2 Institute of Zoology, University of Veterinary Medicine, Bünteweg 17, D-30559 Hannover, Germany; 3 Institute for General Radiology and Medical Physics, University of Veterinary Medicine, Bünteweg 17, D-30559 Hannover, Germany; 4 Landesamt für Natur, Umwelt und Verbraucherschutz Nordrhein-Westfalen (LANUV), Fisheries Ecology, Heinsberger Straße 53, D-57399 Kirchhundem, Albaum Germany

**Keywords:** Carp, Stunning procedures, Animal welfare, Electrical stunning, Visually-evoked responses

## Abstract

**Background:**

Common carp *Cyprinus carpio* is an important food fish in Central Europe, which in some regions is consumed as part of local tradition. The majority of carp are sold by small retailers and not processed in commercial processing plants. The overall objective of this study was to monitor how animal welfare is safeguarded during the stunning and slaughtering of carp for retail sale. For this, the stunning and slaughtering process was monitored on 12 carp farms. Four welfare-related parameters were assessed: (i) stunning success, (ii) injuries related to the applied stunning method, (iii) time between stunning and slaughter, and (iv) visible responses of carp during slaughtering. In addition, indicators of physiological stress were measured. In order to analyse whether the absence of behavioural indicators of consciousness after electrical stunning was correlated with unconsciousness a complementary laboratory study was performed. Here, carp were exposed to electrical current densities between 0.09 and 0.41 A/dm^2^. The presence of behavioural responses and visually-evoked responses (VER) in the electro-encephalogram in response to light flashes as indicators for an absence of consciousness was recorded.

**Results:**

The carp farms applied manual percussive (18%) or electrical (23%) stunning methods, while the majority of farms used a combination of electrical stunning immediately followed by manual percussive stunning (59%). In the latter condition, 92.6% of stunned carp displayed no behavioural indicators of consciousness and significantly fewer injuries related to mishits compared to sole percussive stunning. In the laboratory study, behavioural indicators of consciousness recovered in carp between 1 and 9 min following removal of the electrical current. However, VER could be recorded already at 30 ± 8 s post stunning. This indicates a fast recovery of carp from electrical stunning when exposed to current densities in the range of those generated by commercially available stunning instruments for fish.

**Conclusions:**

Under field conditions, percussion (applied manually) and electrical stunning might be poor inducers of unconsciousness before slaughter, while a combination was most effective. In order to undertake improvements in electrical stunning, further investigations into the current density, required for inducing prolonged insensibility in carp during electrical stunning, are needed.

## Background

The demand for consuming fish is steadily increasing not only because of a growing global population, but also due to consumers preferring fish as a healthy food [1]. The consumers expect high quality fish meat, which is produced with minimal environmental impact, safeguarding animal welfare [2, 3]. Even though farmed fish are often not addressed as animals to care for, but as crops to harvest [4] and the moral status of fish is unresolved, finfish being vertebrates enjoy in Europe the same legal protection as farm animals. Finfish are, for instance, included in the EU Council Regulation (EC 1099/2009) [5] on the protection of animals at the time of killing. This regulation determines that “animals shall be spared any avoidable pain, distress or suffering during their killing …” (Article 3.1EC No. 1099/2009) [5]. According to this regulation, farm “animals shall only be killed after stunning” and “the loss of consciousness and sensibility shall be maintained until the death of the animal” (Article 4, EC No. 1099/2009) [5]. For farmed fish, the European Food Safety Authority (EFSA) issued a scientific opinion on welfare aspects of the principal methods for stunning and killing in 2004 [6] and concluded that “many fish killing processes are designed for commercial efficiency rather than welfare priorities.” Hence, “many existing commercial killing methods expose fish to substantial suffering over a prolonged period of time” [6]. This report [6] further highlighted that because of large differences in “ecological adaptations and evolutionary developments” between farmed fish species, appropriate methods for stunning and killing must be developed and optimised for each species under consideration [6, 7].

One of the major freshwater fish species farmed in mainland Europe is the common carp (*Cyprinus carpio* L., 1758). Common carp is mainly raised in shallow ponds in monoculture or in polyculture with fish from different cyprinid species, which occupy slightly different ecological niches in the pond system [8]. The common carp is regarded as a domesticated species [9] which is well adapted to the husbandry systems it is reared in [10]. In some regions in Central Europe, the consumption of carp during the “carp season”, which lasts from autumn to spring, is part of the local tradition. In these regions, the majority of carp are sold as whole fish on farms, by small retailers, in supermarkets or in restaurants. The majority of fish are killed on demand, only a small proportion of the production is processed in commercial plants [7]. In accordance with EFSA [7], percussion and/ or whole-body electrical stunning in water, followed by evisceration are the main methods used in European carp aquaculture for stunning and killing carp. Percussive stunning is performed for each fish singly with a sharp blow (or some blows) on the head of the carp with a wooden or plastic club (“priest”) [11]. In a risk assessment [7], a mishit or a hit with insufficient force, which does not render fish unconscious, resulting in the fish being further processed while still conscious are considered major hazards associated with percussive stunning. An alternative to percussive stunning could be electrical stunning of fish by passing an electrical current through the fish immersed in tank water by submerged electrodes, or by applying the electrical current to the head of the fish outside the water [11]. Hazards related to electrical stunning include the application of currents and voltages, which are insufficient for rendering fish unresponsive to external stimuli (considered as unconscious) instantaneously [7]. This also would result in continuing with the processing of fish despite it still being responsive to external stimuli, i.e., conscious. In a study on the efficiency of electrical stunning, Lambooij et al. [11] observed that carp exposed to an overall current density of 0.14 A/dm^2^ for 1 s were stunned immediately. Hence, it was concluded that applying electrical current at this density would result in an effective stunning of carp at marketable size (approx. 1–1.5 kg body weight [11]).

To ensure whether the applied stunning method induces a loss of consciousness, it is necessary to recognise whether the fish is in a stage of insensibility. Consciousness can be assessed by electro-encephalogram (EEG) recordings; in particular, responses to stimuli such as visual evoked-responses (VER) or somato-sensory evoked responses [12]. Such sensory-evoked responses indicate that the brain can support the processing of an external sensory stimulus. If an external stimulus can be processed it can be assumed that the brain is capable of supporting consciousness [12]. Possible brainstem/ behavioural indicators of consciousness like the control of body posture, regular operculum movements or eye roll reflex can be used in some fish species in which the behavioural indicators correlate with neural activity [13]. In carp, the presence of these behavioural/ brain stem responses may be used as evidence for consciousness. Nevertheless, it is questioned whether their absence reliably indicates a loss of sensory perception [7]. EEG recordings, however, are only suitable in laboratory settings and cannot be applied on farms. Therefore, adequate training of operators in recognising behavioural indicators of consciousness for safeguarding humane killing of carp is essential [14].

For the German carp aquaculture, no data are available how animal welfare is safeguarded at the time of stunning and killing of farmed carp. Percussion and whole-body electrical stunning in water are prescribed to be applied to farmed carp as stunning methods by the German Regulations for Animal Welfare during Slaughter (Tierschutz-Schlachtverordnung, TierSchlV [15]). In the current pilot study, we analysed for the first time how the stunning and killing process of carp are implemented on carp farms with regard to the applied stunning method, the stunning success and the occurrence of major hazards. In previous laboratory experiments, we analysed whether the absence of behavioural indicators of consciousness correlated with a loss of neural activity.

## Methods

### Laboratory study

A laboratory study was conducted to evaluate whether commercially available stunning instruments generate electrical parameters necessary for a successful electrical stunning of carp. A further aim was to analyse whether a loss of behavioural/ brain stem indicators of consciousness, such as control of body posture, operculum movements and eye roll reflex correlate with a loss of VER as evidence of unconsciousness.

#### Fish and husbandry

Common carp (*Cyprinus carpio L*.; *n* = 56) with size ranging from 28.3 to 37.4 (32.5 ± 2.43) cm and weighing 0.613 to 1.548 (1.085 ± 0.230) kg were collected from a carp farm and kept in groups of two to three individuals in 400 l tanks filled with tap water at ambient temperature for about one week in the laboratory facilities of the Fish Disease Research Unit, University of Veterinary Medicine Hannover, Germany. All animal experiments were performed under approval of the Lower Saxony State Office for Consumer Protection and Food Safety, Germany (LAVES, Oldenburg, Germany) (reference number 09/1714) regarding internationally accepted veterinary standards and federal guidelines.

#### Position of electrodes

For the monitoring of VER, EEG electrodes had to be positioned intracranially over the *cerebellum* and *tectum opticum*. The recording electrode was situated on the right hemisphere of the *tectum opticum*, the electrodes over the *cerebellum* and the left hemisphere of the *tectum opticum* were used as grounding electrodes and to suppress current artefacts [16, 17]. To determine the exact positioning on the skull of these electrodes, in total, 24 carp specimens were euthanised using buffered MS222 (Pharmaq, UK) at a concentration of 0.5 g/l. Subsequently, these carp were either deep frozen and cut longitudinally into1 cm slices or dissected to determine the positions of the *tectum opticum* and *cerebellum* in relation to external features of the head and the variation in these measurements among individuals.

#### Implantation of the electrodes and recording of visual-evoked responses (VER) of the brain

Prior to implanting the electrodes, the carp were anaesthetized using buffered MS222 at a concentration of 0.15 g/l. For maintenance of narcosis a dosage of 0.075–0.100 g/l buffered MS222 was applied to the gills of the carp in a closed water circuit system. For positioning of the electrodes, the exact locations of *cerebellum* and *tectum opticum* were determined using the measurements from the previously dissected carp. The electrodes were fixed to the skull by means of a bonding agent used in dentistry (GULMA comfort bond, Kulzer, Germany). Briefly, above the positioning locations, the skin was removed from the skull until the cranial bone was dorsally exposed in an area of about 2 × 2 cm. The bone was subsequently treated with 20% phosphoric acid (GLUMA Etch 20-Gel) and with the bonding agent GLUMAComfort Bond (Chemicals from Heraeus Kulzer, Germany), which was polymerised by the light of a polymerization lamp (LITEMA, Germany) in order to prepare the skull area for firm attachment of the electrodes and the filling composite (see below). Then, three holes, each 1.5 mm in diameter were drilled into the cranial bone above the *cerebellum* and the two hemispheres of the *tectum opticum.* EEG electrodes (0.4 mm silver plated copper wire (Conrad, Germany) fixed in a 0.9 × 40 mm cannula (Braun, Germany), which was shortened to 1.5 cm and connected to a shielded copper wire (Conrad, Germany), were inserted through the holes and advanced for approximately 1 to 1.5 cm. Thereafter, the electrodes were fixed by a mix of the bonding agent (GLUMA Comfort Bond) and a filling composite (CHARISMA, Heraeus Kulzer, Germany), which then was polymerised using the polymerisation lamp.

To amplify recorded signals, recording and reference electrodes were connected to a differential amplifier (Differential-Amplifier WPI DAM 50; band-pass filter10 Hz to 3 kHz, 1000-fold amplification, World Precision Instrument, USA). To further eliminate irrelevant interfering signals a downstream bandpass filter (Ithaco 4213 Electronic Filter, Ithaco, USA), was set to the same frequency range. Amplified and filtered signal cycles of 116 ms duration were averaged (512 signals) using a digital oscilloscope (Hitachi Digital VC 7504, Hitachi Europe, Germany) in order to reduce background noise as far as possible and to increase the amplitude of the neural signal. Light stimuli were generated by means of a stroboscope (eurolite Action Strobe 300, Conrad, Germany), which was triggered by a pulse generator (HSE Stimulator P, Harvard Apparatus, UK) to generate light pulses of 1.5 ms. The trigger signal was displayed as a rectangular pulse on the second channel of the oscilloscope for temporal correlation of the VER with the light stimulus. During presentation of light stimuli and recording the room was kept dark.

#### Whole body electrical stunning

All carp subjected to electrical stunning were individually equipped with EEG electrodes and placed in a U-shaped adjustable PVC grid or a fenestrated aquarium with plastic grids on the sides and internal adjustable separators in order to restrain possible movements of the carp. Then the carp were placed individually in a polyethylene tank equipped with plate electrodes for applying electrical stunning. In all stunning experiments, the conductivity of water was adjusted to 600 μS/cm and the temperature was kept in the range of the water in the holding tank (16.1–22.7 °C). Two different systems were used for applying the current: In one system, stainless-steel plate-electrodes, each having an area of 22 dm^2^, were placed at a distance of 31 to 34 cm at the top and the bottom (t/b) of a polyethylene tank with a volume of 117 l and a viewing window at the front side. In this tank, carp were fixed with the fenestrated aquarium. In the second system, stainless-steel plate-electrodes, each 9.45 dm^2^, were placed at a distance of 22.0–22.2 cm laterally from the carp in a plastic tank with a volume of 26.7 l. In this tank, the carp were restrained in the flexible U-shaped plastic grid, which was closed at the top and fixed at the upper margin of the tank in order to allow the carp to keep an upright body position.

**Fig. 1:**
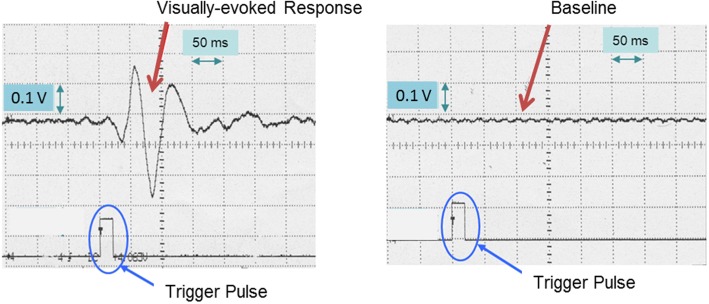
Averaged visually- evoked response (VER, *n* = 512 signals) in the electro- encephalogram of carp. Left panel: Neuronal responses were triggered by light flashes. The onset of the light flash corresponds to the onset of the trigger pulse. Right panel: Averaged control condition without light stimulus

Prior to applying electrical stunning, carp were monitored for any signs of consciousness, such as maintenance of an upright body posture and regular operculum movements. Then, EEG recordings were taken with and without light stimuli to confirm the correct position of the EEG electrodes and whether recorded signals were indeed visually-evoked responses of the carp’s brain (Fig. 1).

Next, the EEG-measuring devices were disconnected from the experimental carp and the plate electrodes were connected to a power supply delivering either a voltage of 50 V (sinusoidal a. c.; constructed by the technical service of LAVES, in order to mimic stunning devices present on carp farms) or 150 V (KT No.8.99, Karl Schermer, Hannover, Germany, modified by the aforementioned technical service for an application on aquaculture farms). Applied voltages and currents were measured with a multimeter (Voltcraft® VC 170, Digital Multimeter, Conrad Electronic, Switzerland) and achieved current densities were calculated by using the following formula:

$$ \mathrm{Current}\ \mathrm{density}\ \left[\frac{\mathrm{A}}{\mathrm{d}{\mathrm{m}}^2}\right]=\mathrm{conductivity}\ \mathrm{of}\ \mathrm{water}\ \left[\frac{\mathrm{S}}{\mathrm{d}\mathrm{m}}\right]\ast \frac{\mathrm{voltage}\ \left[\mathrm{V}\right]}{\mathrm{d}\mathrm{istance}\ \mathrm{of}\ \mathrm{plate}\ \mathrm{electrodes}\ \left[\mathrm{dm}\right]} $$

$$ \mathrm{Amperage}\ \left[\mathrm{A}\right]=\mathrm{current}\ \mathrm{density}\ \left[\frac{\mathrm{A}}{\mathrm{d}{\mathrm{m}}^2}\right]\ast \mathrm{area}\ \mathrm{of}\ \mathrm{aplate}\ \mathrm{electrode}\ \left[\mathrm{d}{\mathrm{m}}^2\right] $$

An electrical alternating voltage of 50 V and 50 Hz was applied for 5 min (time recommended by the aforementioned technical service) with electrodes placed in lateral positions as well as above and below the fish (bottom/ top position). An electrical alternating voltage of 150 V and 50 Hz was applied in experiments with the same electrode positions for 1 min (time set by the instrument). After stunning, the connection to the EEG-measuring devices was immediately re-installed and EEG responses of carp to light flashes were recorded. Hereby, first VER were registered by30 ± 8 s post stunning. The recordings were then continued until 30 min post stunning. The presence or absence of the behavioural indicators vestibulo-ocular reflex **–** (VOR), operculum movement**–** (OM) and righting behaviour – (RB) was recorded for up to 55 min post stun. Furthermore, changes in skin colour were registered after applying the electrical current. All carp were euthanised with buffered MS222 (500 mg/l) after the end of the experiment.

### Field study

To monitor the stunning process of carp under field conditions and to evaluate the stunning success of the different methods used, on 12 carp farms located in the German regions of Bavaria, Saxony and Lower Saxony the processes of stunning and slaughtering were analysed during routine marketing operations. On some of the farms, different methods for stunning and slaughtering were used. Therefore, a total of 17 stunning and killing processes were analysed. For each process, the applied stunning method and, in case of electrical stunning, relevant parameters such as electrical current density, conductivity of the water and application time of the electric current were registered. During and directly after the electrical current flow, behavioural responses were noted and after applying the electrical current, the percentage of carp showing behavioural characteristics of consciousness or reflexes was documented. If percussive stunning was used as a stunning method, it was applied to each carp in turn with two quick blows to the skull using a wooden or plastic club. For percussive stunning, the percentage of carp experiencing mishits (the stroke hitting the skull but not in the brain region) and which displayed behavioural indicators of consciousness after the blow was recorded. For both stunning methods, it was noted whether a stunning procedure was re-applied to insufficiently stunned carp. Finally, the time span between stunning and slaughtering of the carp by exsanguination and/or evisceration was noted.

Furthermore, on each farm, directly after stunning blood samples were collected from the caudal vein of six to ten carp individuals in syringes with lithium-heparinised beads (Sarstedt, Germany) to prevent blood clotting. From the blood samples, the haematocrit was determined immediately using a haematocrit centrifuge. In addition, a part of the blood sample was centrifuged at 600 x *g* at 4 °C for 15 min, the supernatant plasma was collected and transported on ice to the laboratory. There it was frozen at − 80 °C and kept until use for chemical analysis. Blood cortisol levels were measured by solid phase enzyme-linked immunosorbent assay (ELISA, RE52611, IBL International, Germany). Calcium, glucose, lactate, magnesium, potassium, sodium, and total protein, were analysed with an automated blood analyser (ABX Penta 400, Horiba, Kyoto, Japan) as possible stress indicators. The carp were also examined for the occurrence of injuries caused by the stunning procedure.

### Statistical analysis

For statistical analysis the computer program SigmaPlot (Systat Software GmbH, Germany) was used. Normality was tested with a *Shapiro Wilk Test* and homoscedasticity by computing a Spearman’s rank correlation between the absolute values of the residuals and the observed value of the independent variable at *p* < 0.05*.* When variances were considered equal and the data were distributed normally, an ANOVA, followed by Tukey’s post-hoc test was used to detect statistical differences between groups. When the test for normality failed*,* the Kruskal Wallis ANOVA on ranks test, followed by Dunn’s post hoc test, were used for data analysis. Differences between groups were considered as significant at *p* < 0.05.

## Results

### Laboratory study

In order to analyse whether electrical parameters generated by commercially available stunning instruments may induce a loss of behavioural indicators of consciousness and whether this was correlated with an unresponsiveness to external stimuli, carp were subjected to electrical stunning parameters from commercially available stunning units under laboratory conditions and VER were recorded thereafter. The applied electrical parameters, the current density and attained field strengths are listed in Table 1.

**Table 1:** Electrical stunning of carp: achieved electrical parameters

Voltage, position of the plate electrodes, stunning duration	Current density	Electric field strength
	[A/dm^2^]	[V/dm]
50 V, t/b, 5 min	0.09 ± 0.003	15.5 ± 0.39
50 V, lateral, 5 min	0.14 ± 0.00	22.7 ± 0.07
150 V, t/b, 1 min	0.28 ± 0.005	46.9 ± 0.73
150 V, lateral, 1 min	0.41 ± 0.00	68.2 ± 0.00

Conductivity of water: 600 μS/cm; given are mean ± standard deviation

t/b: plate electrodes positioned at top and bottom; lateral: plate electrodes positioned laterally of stunned carp

When the electrical current was applied, carp were motionless, the body muscles were contracted and breathing movements suspended. Immediately after the electrical current was switched off, all carp showed no behavioural indicators of consciousness and weakened muscle contraction. Between 50 s and 10 min after stunning, OM reappeared in 28 out of the 32 stunned specimens. OM reappeared in carp stunned for 5 min at 0.09 A/dm^2^ after 2.3–9.0 min (25–50 percentile: 3–8 min, median 6.0 min), and at 0.14 A/dm^2^, 5.0–10.0 min (25–50 percentile: 6–8 min, median 6.8 min) post stunning. In carp stunned for 1 min at 0.28 A/dm^2^ OM reappeared after 0.8–3.5 (25–50 percentile: 1.0–2.1 min, median 1.6 min) min and at 0.41 A/dm^2^ between 1.0 and 7.0 min (25–50 percentile: 1.5–2.6 min, median 2.0 min, see Table 2). The reappearance of OM was followed by a reoccurrence of the VOR as an additional sign of recovery in 28 out of the 32 stunned specimens between 1 and 25 min (see Table 4) and 19 out of 27 individuals tried to right themselves between 4 and 55 min post stunning. Only four of the 32 stunned carp showed no signs of recovery.

**Table 2:** Temporal recurrence of behavioural characteristics in carp after exposure to electrical current

		Current density and stunning duration			
		0.09 A/dm^2^	0.14 A/dm^2^	0.28 A/dm^2^	0.41 A/dm^2^
		5 min	5 min	1 min	1 min
VER before	n/total	8/8	8/8	8/8	8/8
VER after	n/total	8/8	7/8	8/8	8/8
Survival	n/total	8/8	4/8	8/8	8/8
OM	n/total	8/8	4/8	8/8	8/8
	min-max (min)	2.25–9	5–10	0.83–3.5	1–7
	median (min)	6	6.75	1.6	2
VOR	n/total	8/8	4/8	8/8	8/8
	min-max (min)	6.5–18	8–25	1–10	4–18
	median (min)	8.75	16.25	3.3	11
RB	n/total	4/4	2/8	7/7	6/8
	min-max (min)	15–25	17–25	4–19.5	14–55
	median (min)	19.5	21	6	27

*VER* visually evoked responses, *OM* operculum movements, *VOR* vestibulo-ocular reflex, *RB* righting behaviour

#### Visually-evoked responses

Before applying the electrical current all carp specimens responded to light flashes by generating a visually-evoked response in the EEG. About 30 s after stunning (i.e., the time needed for reconnecting the electrodes to the recording system) a VER could be observed in 31 of the 32 carp specimens. Only from one carp, which was stunned at 0.14 A/dm^2^ for 5 min, could no VER be recorded after applying the current flow. From all carp, VER were obtained before behavioural characteristics, indicating that a recovery could be observed.

### Field study

In the 17 analysed stunning processes, the carp were stunned by percussion in a total of three processes, by electrical-stunning in four processes, and by an initial electrical stunning followed by percussion in ten processes. In ten processes, the carp were electrically stunned in a water bath, in three processes a dry electrical-stunning method was used, and once a combination of dry and water-bath stunning was applied. For electrical-stunning in a water-bath, plate-electrodes were used in six cases, grid electrodes in three cases and bar electrodes in one case. The farmers used commercially available or self-modified stunning devices.

After percussive and electrical stunning, 69.2 and 71.9% of the carp, respectively displayed no behavioural indicators of consciousness (Table 3). When electrical stunning followed by percussion was used, 92.6% of the carp did not show any behavioural indicators of conciousness.

**Table 3:** Stunning and killing of carp: Overview of applied stunning methods, electrical parameters, stunning success and time

		Electrical parameters				Stunning success	Injuries/ mis-hits	Time until exsanguination	Behavioural signs at time of slaughtering
Stunning procedure no.	Number of fish N total: 113	Water conductivity [μS/cm; MIN-MAX]	Voltage [V; MIN-MAX]	Electrical current densities [A/dm^2^; MIN-MAX]	Stunning time (min)	[%; (n/total number of fish)]	[%; (n/total number of fish)]	[minutes; minimum - maximum]	[%; (n/total number of fish)]
Stunning by percussion									
1	5	n.a.	n.a.	n.a.	n.a.	80.0 (4/5)	20.0 (1/5)	0.5–2	60.0 (3/5)
2	2	n.a	n.a	n.a	n.a	100.0 (2/2)	100.0 (2/2)	30	0.0 (0/2)
3	6	n.a	n.a	n.a	n.a	50.0 (3/6)	0.0 (0/6)	0.5–1	50.0 (3/6)
Summary Percussion	13	n.a	n.a	n.a	n.a	69.2 (9/13)	23.1 (3/13)	0.5–30	46.2 (6/13)
Electrical stunning									
4	10	453	47	0.0213	6	90.0 (9/10)	0.0 (0/10)	2	90.0 (1/10)
5	6	337	36	0.0267	2	50.0 (3/6)	50.0 (3/6)	2.5	50.0 (3/6)
6	10	n.a. (Dry stunning)	n.a.	n.a	1	80.0 (8/10)	10.0 (1/10)	1	20.0 (2/10)
7	6	398	36	0.0276	1	50.0 (3/6)	0.0 (0/6)	0.5	50.0 (3/6)
Summary Electrical stunning	32	337–1179	36–47	0.0213–0.0276	1–6	71.9 (23/32)	12.5 (4/32)	0.5–2.5	28.1 (9/32)
Electrical stunning followed by percussion									
8	6	1200	33	0.0759	2	50.0 (3/6)	0.0 (0/6)	0.5–2	66.0 (4/6)
9	10	441	54	0.0238	5	100.0 (10/10)	20.0 (2/10)	30	0.0 (0/10)
10	10	526	39	0.0409	0.5	100.0 (10/10)	20.0 (2/10)	1–3	0.0 (0/10)
11	2	/ (Dry stunning)	/	/	1.5–2	100.0 (2/2)	50.0 (1/2)	1–3	0.0 (0/2)
12	10	587	38	0.0441	0.5	90.0 (9/10)	0.0 (0/10)	0.5–2	10.0 (1/10)
13	6	939	33	0.0808	1	100.0 (6/6)	0.0 (0/6)	0.5–1	16.7 (1/6)
14	6	939	33	0.0808	1	100.0 (6/6)	0.0 (0/6)	2–5	16.7 (1/6)
15	6	939	33	0.0808	1	100.0 (6/6)	0.0 (0/6)	0.5–1	0.0 (0/6)
16	6	822	29	0.1464	2	100.0 (6/6)	0.0 (0/6)	0.5–1	0.0 (0/6)
17	6	/ (Dry stunning)	/	/	1.75	83.3 (5/6)	0.0 (0/6)	2–5	16.7 (1/6)
Summary Electrical stunning followed by percussion	68	441–959	29–54	0.0238–0.1464	0.5–5	92.6 (63/68)	7.4 (5/68)	0.5–30	11.8(8/68)

After percussive stunning, three out of 13 carp (23.1%) showed injuries in paramedian positions of their heads, resulting from mishits. After electrical stunning, four out of 32 fish (12.5%) received external injuries, for instance, during dry electrical stunning, resulting from contact with the electrodes (Table 3).

An overview of the stunning success, the percentage of carp displaying no behavioural indicators of consciousness post stunning, the percentage of carp receiving injuries from the stunning process, of those showing behavioural indicators of consciousness at the time of slaughter, and the time until exsanguination are presented in Table 3.

During wet electrical stunning, stunning tanks were filled with water at an electrical conductivity between 337 and 1200 μS. The stunning devices generated an electrical voltage between 29 and 54 V, which resulted in an electrical current density of between 0.023 and 0.146 A/dm^2^. When considering water conductivity, especially when the conductivity was very low (< 400μS) or very high (> 1000 μS), the percentage of successfully stunned carp was low (50.0%). An overview of water conductivity and electrical parameters achieved during stunning are presented in Table 3.

Most of the carp were killed within 0.5 and 3.0 min after stunning, mainly by evisceration (ten cases), by gill cuts, or destruction of the heart (seven cases).

In order to monitor stress associated with the stunning and killing methods applied on the farms, physiological parameters, including cortisol, glucose, lactate and sodium levels of blood plasma were measured and compared between the applied stunning methods. Mean plasma concentrations of the divalent ions calcium and magnesium ranged between 2.2 and 2.9 mmol l^− 1^ (Ca^2+^) and 1.1 and 1.6 mmol l^− 1^(Mg^2+^) and did not differ between carp after percussive or electrical stunning. Significant differences between the stunning methods were observed in cortisol, glucose, haematocrit and sodium measurements (Table 4). Mean cortisol measurements ranged between 62.1 and 337.7 ng mL^− 1^and were significantly elevated on farms in which carp were stunned by percussion compared to farms, which used electrical stunning or electrical stunning followed by percussion. Likewise, glucose measurements varied to a large extent, with mean farm levels ranging between 2.7 and 16.6 mmol l^− 1^ (Table 4). Plasma glucose levels were significantly lower in carp subjected to electrical stunning compared to carp stunned by percussion, or by electrical stunning followed by percussion (Table 4). Mean plasma sodium levels varied between 115.2 and 151.5 mmol l^− 1^with significantly lower values after electrical stunning compared to percussion, or electrical stunning followed by percussion. Haematocrit values ranged between 25.5 and 41.8% and were significantly lower in carp subjected to percussive stunning compared to electrical stunning, or electrical stunning followed by percussion. Mean lactate levels per farm ranged between 1.0 and 41.8 mmol l^−1^, but did not differ in carp after percussion or electrical stunning (Table 4). A direct correlation between insufficient stunning and high cortisol and glucose levels or decreased sodium and haematocrit measurements (data not shown) could not be detected.

**Table 4:** Clinical chemical parameters in blood of carp after different stunning methods

Stunning procedure	N [number of fish]	Calcium [mmol/l]^c^	Cortisol [ng/mL]^c^	Glucose [mmol/l]	Hematocrit [%]	Lactate [mmol/l]	Magnesium [mmol/l]	Potassium [mmol/l]	Sodium [mmol/l]	Total protein [g/l]
Percussive stunning										
1	5	2.5 ± 0.12	246.9 ± 142.25	7.3 ± 2.48	28.5 ± 5.54	4.8 ± 2.58	1.1 ± 0.21	2.5 ± 0.38	133.0 ± 4.38	27.1 ± 1.94
2	2	2.2 ± 0.38	276.5 ± 7.78	16.6 ± 9.26	25.5 ± 3.53	8.7 ± 1.33	1.5 ± 0.21	2.0 ± 1.10	151.5 ± 13.44	50.5 ± 4.95
3	6	2.4 ± 0.10	193.7 ± 54.18	2.7 ± 1.00	26.8 ± 2.92	5.7 ± 1.93	1.3 ± 0.11	2.5 ± 0.12	136.8 ± 3.37	21.5 ± 4.32
Summary Percussion	13	2.4 ± 0.18^a^	228.3 ± 99.94^a^	6.7 ± 5.63^b^	27.4 ± 4.16^a^	5.8 ± 2.44^a^	1.2 ± 0.21^a^	2.5 ± 0.43^a^	137.3 ± 8.08^b^	28.1 ± 10.40^a^
Electrical stunning										
4	10	2.9 ± 0.20	119.6 ± 82.48	2.6 ± 0.78	38.7 ± 8.88	8.9 ± 2.38	1.3 ± 0.27	1.8 ± 0.40	129.6 ± 8.54	29.4 ± 4.88
5	6	2.3 ± 0.30	91.4 ± 32.94	3.9 ± 0.74	31.9 ± 4.34	1.2 ± 0.42	1.3 ± 0.17	2.7 ± 0.46	136.4 ± 17.32	28.9 ± 6.82
6	10	2.3 ± 0.21	62.1 ± 44.71	3.2 ± 2.10	32.5 ± 3.81	3.5 ± 2.36	1.1 ± 0.18	2.1 ± 0.25	117.2 ± 8.77	24.2 ± 6.23
7	6	2.5 ± 0.16	217.8 ± 35.18	3.4 ± 0.90	33.2 ± 3.19	10.2 ± 2.35	1.4 ± 0–18	5.1 ± 0.93	134.2 ± 0.02	24.0 ± 3.85
Summary Electrical stunning	32	2.5 ± 0.32^a^	114.0 ± 76.54^b^	3.2 ± 1.37^a^	34.4 ± 6.32^b^	5.9 ± 4.12^a^	1.2 ± 0.25^a^	2.7 ± 1.28^a^	128.112.68^a^	26.7 ± 5.94^a^
Electrical stunning followed by percussion										
8	6	2.5 ± 0.15	253.0 ± 72.35	6.0 ± 3.02	35.5 ± 4.28	4.1 ± 2.47	1.1 ± 0.16	3.3 ± 0.32	144.8 ± 8.32	35.0 ± 10.29
9	10	2,6 ± 0.19	263.047.89	5.1 ± 1.10	35.3 ± 4.57	9.0 ± 2.00	1.6 ± 0.24	1.9 ± 0.54	147.0 ± 15.91	28.1 ± 5.30
10	10	2,5 ± 0.33	89.7 ± 29.33	3.7 ± 0.96	31.9 ± 5.25	1.7 ± 1.30	1.2 ± 0.27	5.5 ± 8.82	138.1 ± 12.13	23.7 ± 8.15
11	2	2.4 ± 0.03	62.9 ± 22.45	3.9 ± 1.34	29.5 ± 7.78	1.1 ± 0.26	1.3 ± 0.04	2.7 ± 0.24	143.0 ± 4.24	20.5 ± 2.12
12	10	2.2 ± 0.32	71.6 ± 16.23	2.2 ± 0.35	25.6 ± 2.55	1.0 ± 0.43	1.2 ± 0.08	2.6 ± 0.44	131.3 ± 8.89	23.3 ± 2.50
13	6	2.7 ± 0.20	66.6 ± 59.36	4.3 ± 1.81	37.9 ± 6.24	3.8 ± 1.22	1.4 ± 0.12	2.8 ± 0.62	133.8 ± 2.56	24.2 ± 3.43
14	6	2.6 ± 0.10	81.7 ± 43.78	4.2 ± 0.74	40.2 ± 6.11	6.9 ± 1.67	1.5 ± 0.16	2.8 ± 0.61	133.0 ± 6.84	26.7 ± 2.88
15	6	2.8 ± 0.49	337.7 ± 104.13	6.3 ± 2.23	41.8 ± 9.22	2.8 ± 1.09	1.2 ± 0.14	3.6 ± 0.37	115.5 ± 10.82	34.0 ± 4.15
16	6	2.5 ± 0.07	112.2 ± 36.02	4.4 ± 0.37	38.3 ± 6.32	3.5 ± 0.65	1.4 ± 0.09	3.2 ± 0.54	135.8 ± 2.14	28.3 ± 3.83
17	6	2,2 ± 0.18	135.7 ± 47.36	3.7 ± 1.77	35.3 ± 7.34	3.9 ± 1.05	1.1 ± 0.14	2.6 ± 0.16	117.7 ± 9.71	19.8 ± 3.97
Summary Electrical stunning followed by percussion	68	2.5 ± 0.31^a^	151.32 ± 105.96^b^	4.2 ± 1.83^b^	34.7 ± 7.26^b^	3.9 ± 3.00^a^	1.3 ± 0.23^a^	3.1 ± 3.43^b^	134.3 ± 13.66^b^	26.5 ± 6.86^a^

Mean ± standard deviation

Statistical evaluation was performed on measurements from individual fish in order analyse whether stunning success in individual fish had an impact on clinical chemical parameters of the blood

^a^ measurements of this parameter are not significantly different between farms

^b^ measurements of this parameter are significantly different between farms

^c^ Data distributed normally, statistical evaluation by ANOVA followed by Tukey’s post hoc test. Other parameter: Data distributed not normally, statistical evaluation by ANOVA on ranks followed by Dunn’s post hoc test. Data for a clinical chemical parameter sharing the same superscript letter are not significantly different (total *n* = 113)

## Discussion

The welfare of carp during stunning and slaughtering was considered by EFSA [7] and it was noticed that fewer than 10% of carp for human consumption were processed in commercial processing plants. Instead, most carp were processed in supermarkets, by small retailers or on fish farms. Substantive data on the stunning and killing methods used were not available, but in a questionnaire to EU member states percussion and electrical stunning were reported as being most commonly used [7]. Applying percussive or electrical stunning is also stipulated by the German Regulations for Animal Welfare and Slaughter. Therefore, only these methods or a combination of these two were applied on carp farms visited in the present study. The exact procedures for applying these methods, in particular, parameters for electrical stunning are not stipulated and therefore, the practical application of stunning and killing varied on almost every farm.

Every applied stunning method should render carp unresponsive to external stimuli (considered as unconscious) in order to ensure humane killing [7], and the presence of behavioural responses like operculum movements, eye roll or flight reaction upon touching can be used as evidence of consciousness [16].

Electrical stunning is considered as humane because a fish becomes insensible immediately after exposure to an electrical field provided that sufficient current is administered [11]. Rainbow trout remain motionless and apparently insensible for several seconds even after a 1-s exposure to an electrical field of sufficient field strength. This suggests that the onset of insensibility may occur within less than 1 s [18]. The persistence of insensibility after removal of the electrical field increased with increasing exposure duration [13, 18, 19]. However, the exposure to an electrical field of insufficient field strength can cause paralysis without loss of consciousness. In this state, the fish shows no reflexes or behavioural characteristics, but the perception is not totally disrupted [6, 20–22]. Eels subjected to electrical stunning at a voltage of 50 V for a prolonged period of time turned upside down and stopped breathing for a limited period of time when the electrical current was switched off. This was followed by sluggish behaviour [23]. This observation suggests that in this case applying the electrical current resulted in a phase of exhaustion during which the eels seemed to be unconscious and insensible to external stimuli but were not stunned immediately. Hence, this treatment was considered to be painful for the eels [23]. The study on electrical stunning in eels [23] indicated that the absence of behavioural traits and signs of recovery might not be sufficient for an assessment of insensibility in fish. Instead, EEG recordings, in particular the observation of evoked responses, should be applied [6, 20, 22, 23]. This, however, is only applicable under laboratory conditions. Therefore, in the present study, laboratory experiments were performed to confirm whether the absence of behavioural responses of carp would correlate with unresponsiveness to external stimuli, which then could be considered as unconsciousness. Carp were exposed to an electric field generated by commercially available stunning devices and VER of the brain and signs of recovery were recorded after removing the electric field. With a stunning device operating at 50 V, current densities of 0.09 and 0.14 A/dm^2^ were achieved and with a device operating at 150 V current densities of 0.28 und 0.41 A/dm^2^ were achieved, both at a water conductivity of 600 μS/cm. In all cases, prolonged stunning induced a loss of righting behaviour, OM and VOR, but VERs were present in all carp at the time of recording, i.e., at about 30 s after removing the electric field. In a previous study, epileptiform insults could be recorded in EEGs of carp immediately after electrical stunning with a current density of 0.14 or 0.73 A/dm^2^ [11]. In this state, carp were considered to be unconscious and insensible [22, 24, 25]. After applying 0.14 A/dm^2^ for 1 s, epileptiform insults could be recorded for 34 ± 10 s. Carp recovered and started responding to painful stimuli 30 s to 10 min after the application [11]. The presence of epileptiform insults in the EEG of carp after stunning suggests that at 0.14 A/dm^2^ a stage of insensibility could be induced. In our experiments, EEG electrodes had to be disconnected during exposure of carp to the electric field and were reconnected immediately after the electrical current was switched off. Therefore, the recording of brain activity could only be started about 30 s after stunning. At this time, VER to light stimuli could already be recorded from carp exposed to current densities of 0.14 to 0.41 A/dm^2^. In rainbow trout, the duration of insensibility could be extended by increasing the current magnitude or the duration of exposure [13, 18]. In our experiments, the reoccurrence of behavioural indicators of consciousness was influenced by the duration of exposure, but not the recovery of VER. Our findings could be interpreted as follows: Namely, that insensibility, if at all, was induced for a short period of time and the carp recovered at some point within 30 s. The temporary absence of behavioural indicators of consciousness might then have resulted from exhaustion due to a prolonged duration of stunning. Hence, on farms, recognition of unconsciousness is extremely difficult in carp since the absence of the behavioural traits operculum movement or eye roll reflex do not necessarily indicate a loss of consciousness as result of an effective stunning. Nevertheless, if these behavioural traits can be observed in carp after a stunning operation, these carp have certainly not been stunned.

On several farms, where carp were stunned either by percussion or by electrical stunning alone, these behavioural traits were still observed in some individuals after the stunning procedure or even during slaughter, which represents a compromised welfare for the fish. In a risk assessment of stunning methods, EFSA [7] listed mishits at the wrong place or with too little force and subsequent processing of fish still during consciousness as possible hazards during percussive stunning. This could be substantiated by our observations on farms (present study), where about 23% of the carp showed signs of hits at a wrong place on the head and were processed while showing behavioural signs. For electrical stunning, exposure to insufficient current and subsequent processing still during consciousness, were estimated as serious hazards by EFSA experts [7] and could also be confirmed during our field study. In contrast, in the majority of carp stunned by a combination of electrical stunning followed by percussion, behavioural indicators of consciousness were not observed. Nevertheless, in an overall assessment of this method, a relatively high proportion of carp, that of 7.4%, still displayed operculum movements, which have to be interpreted as signs of consciousness. However, in seven out of the ten stunning and killing processes in which this stunning method was applied, behavioural indicators of consciousness were absent in all carp. This underlines the importance of an adequate training of operators in performing the stunning and recognising behavioural indicators of consciousness in order to safeguard humane slaughter of carp.

Plasma electrolytes, cortisol and glucose levels were monitored as possible indicators for stress during the stunning and killing process. In particular, measurements of cortisol, glucose and sodium levels varied to a great extent between individuals processed with a particular stunning and killing procedure. When the different procedures were considered, cortisol levels were higher in carp stunned by percussion, while glucose and sodium levels were lower in carp stunned by electrical stunning. Normal cortisol levels in carp are between 5 and 15 ng/ml [26]. Elevated cortisol and glucose levels were found in carp after a stress event [27]. Therefore, the data presented here might indicate that stunning by percussion could be more stressful for carp. However, the wide variations in these measurements between the assessed individual processes underlines the importance of the pre-slaughter process on animal welfare in addition to the stunning method. The handling of carp before stunning differed greatly on the investigated farms, and most likely, the differences in cortisol, glucose or sodium levels were influenced by hazards related to the pre-slaughter process in addition to the stunning method. This view could be supported by our observation that the increase in cortisol levels was not correlated to lower stunning success on farms.

The technical devices used for electrical stunning on the farms differed widely in design and generated electrical parameters. Most farmers used commercially available stunning devices, which were specified for “stunning of fish”. During stunning of carp in a water bath, the achieved current densities varied between 0.016 and 0.146 A/dm^2^. In several of the settings the current densities were lower than 0.1 A/dm^2^, a current density which was recommended for successful stunning of rainbow trout [13, 17]. At lower current densities, operculum movements recovered in rainbow trout between 30 and 50 s post stunning [18]. On several farms, the interval between stunning and slaughter was longer than 30–50 s, which also could explain why in some carp individuals signs of consciousness could be observed at the time of slaughtering. On the majority of farms, recovery of carp before slaughter was prevented by a combination of electrical stunning and percussion. In addition, on these farms, recovered carp were re-stunned by percussion, which might indicate a high staff awareness of good professional practice in the stunning and slaughtering process.

## Conclusion

Our laboratory study showed that after electrical stunning with devices operating at 50 V, carp are unconscious for only a very short period of time (max. 30 s), if at all. Any operculum movement of a carp therefore has to be rated as a sign of perceptiveness. Our field observations indicate that under farm conditions, both percussive and electrical stunning of carp require careful attention. Farmers have to be aware of behavioural signs of consciousness and to re-stun carp by percussion as soon as indicators such as regular operculum movements or the vestibulo-ocular reflex are visible after a stunning method has been applied. As many farmers were aware of this difficulty, an initial electrical stunning and a subsequent re-stunning of carp by percussion were implemented into the work flow on several of the visited farms. With this procedure, the farmers could ensure that carp were not showing behavioural traits of consciousness at the time of slaughter. As even carp showing no signs of behavioural traits might be perceptive shortly after stunning, slaughtering of carp should be performed immediately afterwards.

Conditions for electrical stunning need to be optimised. In particular, investigations into the current density, which is required for inducing insensibility and determining how the period of insensibility could be increased to more than a few seconds, are needed.
